# Distribution and antimicrobial susceptibility profiles of bacterial isolates from vaginal discharge specimens among hospitalized patients in a tertiary maternal and child healthcare hospital in Shenzhen, China

**DOI:** 10.3389/fmicb.2026.1849684

**Published:** 2026-06-03

**Authors:** Tongyan Ding, Xiaochun Liu, Shaoxiang Lin, Xiuju Liu, Xiaojie Zhou, Zhimin Zhao, Shujuan Yang, Futing Liao, Qiaoxin Zhang, Zhenwen Zhou

**Affiliations:** 1 Clinical Laboratory, Key Laboratory of Bacterial Antimicrobial Resistance and Prevention, Medical Research Institute of Maternal and Child, Longgang District Maternity & Child Healthcare Hospital of Shenzhen City (Affiliated Shenzhen Women and Children's Hospital (Longgang) of Shantou University Medical College), Shenzhen, Guangdong, China; 2 Clinical Laboratory, Guangzhou Women and Children’s Medical Center, Guangdong Provincial Clinical Research Center for Laboratory Medicine, Guangzhou Medical University, Guangzhou, Guangdong, China; 3 The First Affiliated Hospital of Shantou University Medical College, Shantou, Guangdong, China

**Keywords:** antimicrobial susceptibility, bacterial isolates, hospitalized patients, maternal and child healthcare hospital, vaginal discharge

## Abstract

**Introduction:**

Abnormal vaginal discharge is a common and clinically important presenting complaint and reason for specimen submission, often suggesting lower genital tract disorders such as vaginal infections, and may adversely affect women’s reproductive health and pregnancy outcomes. This study aimed to investigate the distribution and antimicrobial susceptibility profiles of common bacteria isolated from vaginal discharge specimens submitted from hospitalized patients at Longgang District Maternity & Child Healthcare Hospital of Shenzhen City, in order to provide a reference for the interpretation of clinical specimen results and empirical antimicrobial therapy.

**Methods:**

Non-duplicate bacterial isolates recovered from vaginal discharge specimens collected from hospitalized patients between January 1, 2021 and December 31, 2025 were retrospectively analyzed, and the composition of common vaginal bacterial isolates, the age distribution of patients, and the antimicrobial susceptibility profiles of major isolates to commonly used antibiotics were assessed.

**Results:**

A total of 1,094 non-duplicate positive bacterial isolates were identified during 2021–2025, with Gram-positive bacteria predominating (65.2%). The major isolates were *Streptococcus agalactiae*, *Escherichia coli*, and *Klebsiella pneumoniae subsp. pneumoniae*. Most cases were from the obstetrics department, and the majority of patients were women aged 25–34 years. Antimicrobial susceptibility testing showed marked differences in antimicrobial susceptibility profiles among bacterial species: *Streptococcus agalactiae* remained highly susceptible to penicillins and vancomycin, but showed high resistance rates to clindamycin and tetracycline; *Escherichia coli* and *Klebsiella pneumoniae subsp. pneumoniae* had relatively high rates of extended-spectrum β-lactamase (ESBL) positivity, while remaining highly susceptible to amikacin, carbapenems, and tigecycline; some isolates also exhibited notable resistance to trimethoprim-sulfamethoxazole, fluoroquinolones, and certain β-lactam antibiotics.

**Discussion:**

Vaginal bacterial isolates from hospitalized patients were predominantly composed of *Streptococcus agalactiae* and members of the Enterobacteriaceae family, and were mainly derived from the obstetrics department and women of reproductive age. Marked differences in antimicrobial susceptibility profiles were observed among bacterial species, suggesting that maternal and child healthcare hospitals should strengthen local surveillance of isolate distribution and antimicrobial susceptibility in order to support more precise empirical therapy and antimicrobial stewardship.

## 1 Introduction

In gynecological and maternal-child healthcare practice, abnormal vaginal discharge is commonly associated with vaginal infections and other lower genital tract disorders. Among these conditions, aerobic vaginitis (AV), which is associated with the overgrowth of aerobic bacteria, has received increasing attention in recent years (Aklilu et al., 2024; Mishra et al., 2025; Zarmakoupi et al., 2024). Such infections not only cause symptoms such as abnormal discharge and vulvovaginal discomfort, thereby affecting women’s quality of life and reproductive health, but may also further increase the risk of ascending genital tract infections, including cervicitis, endometritis, and pelvic inflammatory disease, and are associated with adverse pregnancy and perinatal outcomes such as miscarriage, preterm birth, premature rupture of membranes, and neonatal infection (Cheng et al., 2025; Dos Anjos Borges et al., 2023). Therefore, vaginal infection is not merely a localized inflammatory condition, but an important clinical problem closely related to women’s reproductive health and maternal–infant outcomes. Standardized laboratory evaluation is thus of considerable importance for clinical assessment and appropriate intervention (Milford et al., 2025).

The vaginal niche is a dynamic micro-ecological environment, and its microbial composition is influenced by multiple factors, including age, hormonal status, pregnancy status, underlying diseases, hygiene habits, and antibiotic exposure (Dube-Zinatelli et al., 2024; Eslami et al., 2024). Once the local micro-ecological balance is disrupted, opportunistic microorganisms or exogenous pathogens may proliferate abnormally, thereby leading to infection. Previous studies have shown that the bacterial isolate profile in submitted vaginal discharge specimens is highly heterogeneous. Common isolates include *Streptococcus agalactiae*, *Escherichia coli*, *Klebsiella pneumoniae*, *Enterococcus* spp., and *Staphylococcus* spp.; however, the predominant species composition and detection rates vary across countries, regions, hospitals, and populations (Hussein et al., 2024). These findings indicate that the vaginal isolate profile has marked regional and population-specific characteristics, and that data from other institutions or regions cannot fully substitute for local evidence.

In clinical practice, empirical treatment for vaginal infections is common. However, as differences in antimicrobial susceptibility profiles among common isolates become increasingly evident, empirical therapy based solely on symptoms may lead to suboptimal efficacy, increased recurrence, and inappropriate antibiotic use (Michalow et al., 2024; Said et al., 2025). Previous studies have suggested that different vaginal isolates show substantial differences in susceptibility to commonly used antimicrobial agents. Some Gram-positive cocci and Gram-negative bacilli have already exhibited high resistance rates to tetracyclines, trimethoprim-sulfamethoxazole, ampicillin, and certain cephalosporins, and multidrug-resistant phenotypes may also occur (Aklilu et al., 2024; Shimeles et al., 2025). Antimicrobial susceptibility testing remains an important basis for evaluating bacterial resistance and guiding rational antibiotic use (Pierce et al., 2023). Therefore, continuous surveillance of the distribution and resistance characteristics of vaginal isolates is of practical value for improving the precision of empirical therapy, optimizing antibiotic use strategies, and strengthening hospital antimicrobial stewardship.

The patient population served by maternal and child healthcare specialty hospitals differs substantially from that of general hospitals, as these institutions primarily care for women of reproductive age, pregnant women, and other perinatal populations, whose bacterial characteristics and clinical medication needs are distinctive (Nguyen et al., 2025; Romero et al., 2023; Yao et al., 2025). On the one hand, physiological changes during pregnancy and the perinatal period may affect the vaginal microenvironment and pathogen colonization characteristics (Xie Y. et al., 2025; Xie Z. et al., 2025). On the other hand, anti-infective treatment must also take into account the safety of the mother, fetus, and newborn, which makes maternal and child healthcare hospitals more dependent on local pathogen surveillance data (Banerjee and Biswas, 2025). In hospitalized patients in particular, previous antibiotic exposure, underlying diseases, hospital exposure, and medical procedures may further influence isolate composition and resistance patterns. Therefore, surveillance of vaginal isolates in hospitalized patients at maternal and child healthcare specialty hospitals may better reflect the real in-hospital infectious microbiological characteristics and treatment needs than studies conducted in outpatient settings.

At present, studies on the distribution and antimicrobial susceptibility profiles of vaginal isolates in China have mainly focused on patients from general gynecology clinics or general hospitals, whereas systematic data on hospitalized patients in maternal and child healthcare specialty hospitals remain relatively limited. As a city with a young and highly mobile population, Shenzhen may have region-specific characteristics in the disease spectrum and infection profile of maternal and child populations; however, local surveillance data on the distribution and antimicrobial susceptibility profiles of vaginal isolates among hospitalized patients remain insufficient. Therefore, this study retrospectively analyzed vaginal isolates from hospitalized patients at a tertiary maternal and child healthcare hospital in Shenzhen, with the aim of clarifying the distribution characteristics and antimicrobial susceptibility profiles of common vaginal isolates and providing local laboratory evidence for clinical practice and rational antibiotic use.

## 2 Materials and methods

### 2.1 Study design

This was a single-center retrospective study. Bacterial culture and antimicrobial susceptibility testing data were collected from vaginal discharge specimens submitted from hospitalized patients at Longgang District Maternity & Child Healthcare Hospital of Shenzhen City between January 1, 2021 and December 31, 2025. Vaginal discharge specimens that underwent bacterial isolation and identification during the study period were included. To avoid duplicate counting, when the same bacterium was isolated from repeated specimens submitted from the same patient within a short period, only the first isolate was retained. This study used only anonymized laboratory data generated during routine clinical practice, and no additional patient intervention or supplementary sampling was performed. The study protocol was submitted to and approved by the Research Project Ethics Committee of the Longgang District Maternity & Child Healthcare Hospital of Shenzhen City (Approval No. LGFYKYXMLL-2026-2).

### 2.2 Participant enrolment

Vaginal discharge specimens that underwent bacterial isolation and identification during the study period were screened. The inclusion criteria were as follows: (1) specimens were collected from hospitalized patients; (2) bacterial isolation and identification were performed; and (3) positive bacterial culture results with available species identification were obtained. The exclusion criteria were as follows: (1) repeated isolation of the same bacterial species from the same patient during the same clinical episode, for which only the first isolate was retained; (2) records with incomplete key information for species identification or antimicrobial susceptibility testing; and (3) non-bacterial pathogens or organisms not recovered by routine bacterial culture. The study population was not categorized according to pregnancy status, marital status, or neonatal outcomes; patient characteristics were summarized mainly by age and clinical department.

### 2.3 Data collection

Bacterial identification and antimicrobial susceptibility testing were performed using the VITEK 2 Compact automated microbiology system (bioMérieux, France). Antimicrobial susceptibility results were interpreted according to the Clinical and Laboratory Standards Institute (CLSI) Performance Standards for Antimicrobial Susceptibility Testing, M100-Ed35 (2025). According to the testing panels available for different bacterial species, the resistance rates, susceptibility rates, and intermediate rates of major clinically relevant bacterial isolates to commonly used antimicrobial agents were recorded.

Laboratory quality control was conducted in accordance with the CLSI M100-Ed35 (2025) standards. Standard reference strains, including *Escherichia coli* ATCC 25922, *Klebsiella pneumoniae* ATCC 700603, *Staphylococcus aureus* ATCC 25923, *Enterococcus faecalis* ATCC 29212, and *Streptococcus pneumoniae* ATCC 49619, were employed for quality control throughout bacterial identification and antimicrobial susceptibility testing to ensure the accuracy and reproducibility of the results.

The following data were collected: the composition and distribution characteristics of common bacterial isolates in vaginal specimens, the age and departmental distribution of patients, antimicrobial susceptibility testing results for major isolates, and defined resistance phenotypes where applicable.

In this study, “antimicrobial susceptibility testing results” refer to categorical results for individual antimicrobial agents, including susceptible, intermediate, and resistant categories. The term “antimicrobial resistance” was used only when describing resistance rates to specific agents or defined resistance phenotypes. Extended-spectrum β-lactamase (ESBL) positivity was assessed for *Escherichia coli* and *Klebsiella pneumoniae subsp. pneumoniae* where applicable. ESBL positivity was determined according to the phenotypic ESBL results generated by the VITEK 2 Compact system and interpreted according to CLSI M100 criteria. Methicillin resistance in *Staphylococcus aureus* was inferred from cefoxitin screening results, and cefoxitin-screen-positive isolates were considered phenotypic methicillin-resistant *Staphylococcus aureus* (MRSA).

### 2.4 Data analysis

Descriptive analysis was primarily performed in this study. The distribution of bacterial species, department composition, age composition, antimicrobial susceptibility testing results, and defined resistance phenotypes were expressed as numbers of isolates and percentages. For bacterial species with a small number of isolates, antimicrobial susceptibility testing results were described for reference only. No subgroup analysis according to pregnancy status, marital status, or neonatal outcomes was performed.

## 3 Results

### 3.1 Bacterial distribution

#### 3.1.1 Major bacterial composition

A total of 1,094 non-duplicate bacterial isolates were identified from submitted vaginal discharge specimens during 2021–2025. Overall, Gram-positive bacteria predominated, accounting for 713 isolates (65.2%), whereas Gram-negative bacteria accounted for 381 isolates (34.8%). Among all isolates, *Streptococcus agalactiae* was the most common species, with 653 isolates (59.7%), followed by *Escherichia coli* with 258 isolates (23.6%), *Klebsiella pneumoniae subsp. pneumoniae* with 64 isolates (5.9%), *Proteus mirabilis* with 41 isolates (3.7%), *Staphylococcus aureus* with 36 isolates (3.3%), and *Enterococcus faecalis* with 10 isolates (0.9%). Other Gram-positive and other Gram-negative bacteria accounted for 1.3 and 1.6%, respectively (Table 1).

**Table 1:** Composition of bacterial isolates from vaginal discharge specimens from 2021 to 2025 (*n*, %).

Organism	2021 (*n* = 176)		2022 (*n* = 214)		2023 (*n* = 211)		2024 (*n* = 216)		2025 (*n* = 277)		Total (*n* = 1,094)	
	*n*	%	*n*	%	*n*	%	*n*	%	*n*	%	*n*	%
Gram-positive bacteria	131	74.4	164	76.6	130	61.6	134	62.0	154	55.6	713	65.2
*Streptococcus agalactiae*	123	69.9	154	72.0	118	55.9	120	55.6	138	49.8	653	59.7
*Staphylococcus aureus*	6	3.4	6	2.8	6	2.8	7	3.2	11	4.0	36	3.3
*Enterococcus faecalis*	0	0.0	3	1.4	0	0.0	4	1.9	3	1.1	10	0.9
Other Gram-positive bacteria	2	1.1	1	0.4	6	2.8	3	1.4	2	0.7	14	1.3
Gram-negative bacteria	45	25.6	50	23.4	81	38.4	82	38.0	123	44.4	381	34.8
*Escherichia coli*	24	13.6	34	15.9	54	25.6	54	25.0	92	33.2	258	23.6
*Klebsiella pneumoniae subsp. pneumoniae*	7	4.0	9	4.2	15	7.1	19	8.8	14	5.1	64	5.9
*Proteus mirabilis*	14	8.0	6	2.8	7	3.3	5	2.3	9	3.2	41	3.7
Other Gram-negative bacteria	0	0.0	1	0.5	5	2.4	4	1.9	8	2.9	18	1.6

Year-by-year analysis showed that Gram-positive bacteria accounted for a relatively high proportion in 2021 and 2022, representing 74.4 and 76.6% of isolates, respectively, whereas Gram-negative bacteria accounted for 25.6 and 23.4%, respectively. Since 2023, the proportion of Gram-negative bacteria increased to 38.4, 38.0, and 44.4% in 2023, 2024, and 2025, respectively, although it remained lower than that of Gram-positive bacteria overall. Meanwhile, the proportion of Gram-positive bacteria showed a gradual downward trend, decreasing from 74.4% in 2021 to 55.6% in 2025.

With regard to the annual distribution of major species, *Streptococcus agalactiae* ranked first in all study years, but its constituent proportion showed a declining trend, decreasing from 69.9% in 2021 to 49.8% in 2025. In contrast, the proportion of *Escherichia coli* generally increased from 13.6% in 2021 to 33.2% in 2025, suggesting a gradual rise in its detection rate among bacteria isolated from vaginal discharge specimens. The proportion of *Klebsiella pneumoniae subsp. pneumoniae* remained relatively stable across the study period, ranging from 4.0 to 8.8%. *Proteus mirabilis* accounted for a relatively high proportion in 2021 (8.0%), followed by a decline and stabilization at a lower level thereafter. *Staphylococcus aureus* and *Enterococcus faecalis* were detected at low frequencies overall, with only minor fluctuations across the years (Table 1).

#### 3.1.2 Departmental and age distribution

**Figure 1:**
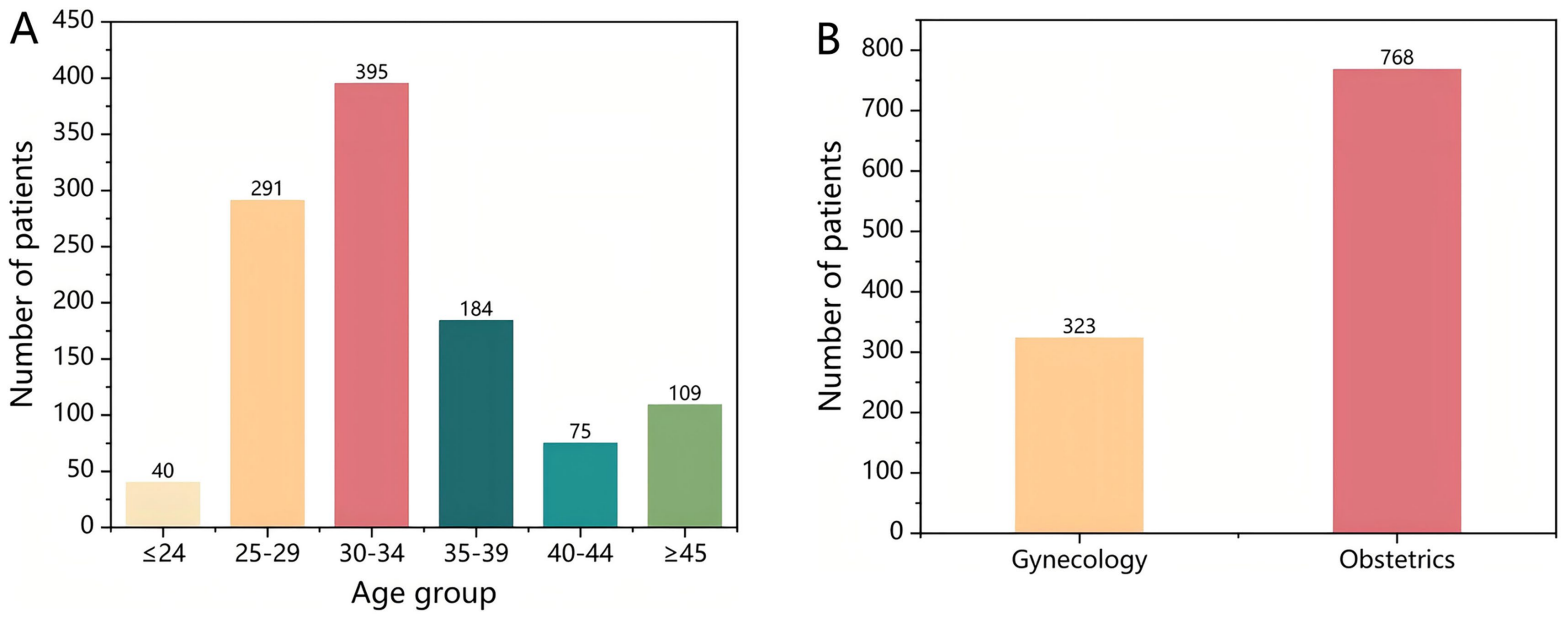
Age and departmental distribution of patients with positive vaginal cultures. (**A**) Age distribution. (**B**) Departmental distribution.

Analysis of the departmental source and age distribution of patients with positive vaginal bacterial cultures showed that positive specimens were mainly from the obstetrics and gynecology departments, including 768 obstetric patients (70.2%), 323 gynecologic patients (29.5%), and 3 patients from other departments (0.3%) (Figure 1B). Overall, vaginal bacterial isolates were predominantly from the obstetric population.

With regard to age distribution, the 30-34-year age group had the largest number of patients, with 395 cases (36.1%), followed by the 25-29-year group with 291 cases (26.6%) and the 35-39-year group with 184 cases (16.8%). The ≥45-year and 40-44-year groups comprised 109 cases (10.0%) and 75 cases (6.9%), respectively, whereas the ≤24-year group had the fewest patients, with only 40 cases (3.7%) (Figure 1A). Overall, patients with positive vaginal cultures were mainly concentrated in the 25-34-year age range, accounting for a total of 686 cases (62.7%), indicating that women of reproductive age, particularly those aged 25–34 years, constituted the main population represented in this study.

### 3.2 Antimicrobial susceptibility profiles

#### 3.2.1 Streptococcus agalactiae

*Streptococcus agalactiae* remained highly susceptible to most commonly used antimicrobial agents. The results showed that the susceptibility rates to ampicillin, linezolid, penicillin, tigecycline, and vancomycin were all 100.0%, while the susceptibility rate to quinupristin/dalfopristin was 99.7%, indicating that these agents still retained good *in vitro* activity against *Streptococcus agalactiae* (Table 2). The highest resistance rate was observed for clindamycin (98.6%), followed by tetracycline (81.0%). In addition, a certain degree of resistance was also observed to moxifloxacin, levofloxacin, and nitrofurantoin, with resistance rates of 20.0, 19.9, and 15.0%, respectively. The intermediate rate for levofloxacin was 1.1%, whereas no intermediate results were observed for the other agents.

**Table 2:** Antimicrobial susceptibility profiles of *Streptococcus agalactiae* to commonly used antimicrobial agents.

Antimicrobial agents	Resistance rate (%)	Susceptibility rate (%)	Intermediate rate (%)
Ampicillin	0.0	100.0	0.0
Nitrofurantoin	15.0	85.0	0.0
Clindamycin	98.6	1.4	0.0
Quinupristin/dalfopristin	0.3	99.7	0.0
Linezolid	0.0	100.0	0.0
Moxifloxacin	20.0	80.0	0.0
Penicillin	0.0	100.0	0.0
Tetracycline	81.0	19.0	0.0
Tigecycline	0.0	100.0	0.0
Vancomycin	0.0	100.0	0.0
Levofloxacin	19.9	79.0	1.1

#### 3.2.2 Staphylococcus aureus

*Staphylococcus aureus* showed substantial variation in resistance to different antimicrobial agents. The results showed that the highest resistance rate was observed for penicillin (63.9%), followed by tetracycline (26.7%) and cefoxitin (screen) (22.2%), suggesting that some isolates may carry a risk of methicillin resistance (Table 3). In addition, the resistance rates to erythromycin and clindamycin were both 13.9%, while levofloxacin showed a resistance rate of 5.6% and an intermediate rate of 2.7%. Overall, *Staphylococcus aureus* remained 100.0% susceptible to gentamicin, vancomycin, linezolid, and teicoplanin, and also showed high susceptibility to trimethoprim-sulfamethoxazole, with a susceptibility rate of 97.2%.

**Table 3:** Antimicrobial susceptibility profiles of *Staphylococcus aureus* to commonly used antimicrobial agents.

Antimicrobial agents	Resistance rate (%)	Susceptibility rate (%)	Intermediate rate (%)
Cefoxitin (screen)	22.2	77.8	0.0
Penicillin	63.9	36.1	0.0
Erythromycin	13.9	86.1	0.0
Clindamycin	13.9	86.1	0.0
Levofloxacin	5.6	91.7	2.7
Gentamicin	0.0	100.0	0.0
Trimethoprim-sulfamethoxazole	2.8	97.2	0.0
Tetracycline	26.7	73.3	0.0
Vancomycin	0.0	100.0	0.0
Linezolid	0.0	100.0	0.0
Teicoplanin	0.0	100.0	0.0

#### 3.2.3 Enterococcus faecalis

*Enterococcus faecalis* remained generally highly susceptible to most commonly used antimicrobial agents. The results showed that the susceptibility rates to ampicillin, high-level streptomycin, linezolid, penicillin, tigecycline, teicoplanin, and vancomycin were all 100.0%, while the susceptibility rate to high-level gentamicin was 90.0%, indicating that these agents still retained good *in vitro* activity against *Enterococcus faecalis* in the present study (Table 4). The highest resistance rate was observed for tetracycline (66.7%), followed by erythromycin (50.0%), which also showed an intermediate rate of 40.0%. The resistance rate to levofloxacin was 20.0%, and that to high-level gentamicin was 10.0%.

**Table 4:** Antimicrobial susceptibility profiles of *Enterococcus faecalis* to commonly used antimicrobial agents.

Antimicrobial agents	Resistance rate (%)	Susceptibility rate (%)	Intermediate rate (%)
Ampicillin	0.0	100.0	0.0
High-level streptomycin	0.0	100.0	0.0
High-level gentamicin	10.0	90.0	0.0
Erythromycin	50.0	10.0	40.0
Linezolid	0.0	100.0	0.0
Penicillin	0.0	100.0	0.0
Tetracycline	66.7	33.3	0.0
Tigecycline	0.0	100.0	0.0
Teicoplanin	0.0	100.0	0.0
Vancomycin	0.0	100.0	0.0
Levofloxacin	20.0	80.0	0.0

#### 3.2.4 Escherichia coli

*Escherichia coli* was the most common Gram-negative isolate in this study, with an ESBL positivity rate of 40.7%. Antimicrobial susceptibility testing showed that *Escherichia coli* was completely susceptible to tigecycline, with a susceptibility rate of 100.0%, and also remained highly susceptible to amikacin, ertapenem, and imipenem, each with a susceptibility rate of 99.2% (Table 5). In addition, the susceptibility rate to piperacillin/tazobactam was 96.5%. The highest resistance rate was observed for trimethoprim-sulfamethoxazole (45.4%), followed by ceftriaxone (40.7%), ampicillin/sulbactam (35.5%), ciprofloxacin (26.3%), aztreonam (23.8%), and cefepime (21.2%). The resistance rate to amoxicillin/clavulanic acid was 4.6%, but the intermediate rate was 13.2%; the intermediate rates for ceftazidime and ampicillin/sulbactam were 9.6 and 22.6%, respectively.

**Table 5:** Antimicrobial susceptibility profiles of *Escherichia coli* to commonly used antimicrobial agents.

Antimicrobial agents	Resistance rate (%)	Susceptibility rate (%)	Intermediate rate (%)
Amikacin	0.8	99.2	0.0
Amoxicillin/clavulanic acid	4.6	82.2	13.2
Ampicillin/sulbactam	35.5	41.9	22.6
Piperacillin/tazobactam	3.1	96.5	0.0
Ceftriaxone	40.7	59.3	0.0
Ceftazidime	10.8	79.6	9.6
Cefepime	21.2	78.8	0.0
Aztreonam	23.8	76.2	0.0
Ertapenem	0.4	99.2	0.0
Imipenem	0.4	99.2	0.0
Ciprofloxacin	26.3	73.7	0.0
Trimethoprim-sulfamethoxazole	45.4	54.6	0.0
Tigecycline	0.0	100.0	0.0

#### 3.2.5 Klebsiella pneumoniae subsp. pneumoniae

*Klebsiella pneumoniae subsp. pneumoniae* was one of the common Gram-negative isolates in this study, with an ESBL positivity rate of 31.8%. Antimicrobial susceptibility testing showed that this organism remained 100.0% susceptible to amikacin, ertapenem, imipenem, and tigecycline. It also showed relatively high susceptibility rates to piperacillin/tazobactam, aztreonam, and ceftazidime, at 91.9, 88.2, and 81.3%, respectively. The highest resistance rate was observed for ampicillin/sulbactam (43.8%), followed by ceftriaxone (31.8%), ciprofloxacin (29.4%), cefepime (23.8%), trimethoprim-sulfamethoxazole (20.3%), and amoxicillin/clavulanic acid (10.6%) (Table 6). The intermediate rates for amoxicillin/clavulanic acid, piperacillin/tazobactam, and ceftazidime were 10.6, 1.6, and 1.5%, respectively.

**Table 6:** Antimicrobial susceptibility profiles of *Klebsiella pneumoniae subsp. pneumoniae* to commonly used antimicrobial agents.

Antimicrobial agents	Resistance rate (%)	Susceptibility rate (%)	Intermediate rate (%)
Amikacin	0.0	100.0	0.0
Amoxicillin/clavulanic acid	10.6	78.8	10.6
Ampicillin/sulbactam	43.8	56.2	0.0
Piperacillin/tazobactam	6.5	91.9	1.6
Ceftriaxone	31.8	68.3	0.0
Ceftazidime	17.2	81.3	1.5
Cefepime	23.8	76.2	0.0
Aztreonam	11.8	88.2	0.0
Ertapenem	0.0	100	0.0
Imipenem	0.0	100	0.0
Ciprofloxacin	29.4	70.6	0.0
Trimethoprim-sulfamethoxazole	20.3	79.7	0.0
Tigecycline	0.0	100.0	0.0

#### 3.2.6 Proteus mirabilis

*Proteus mirabilis* remained generally highly susceptible to most commonly used antimicrobial agents (Table 7). The results showed that the susceptibility rates to amikacin, amoxicillin/clavulanic acid, piperacillin/tazobactam, and ertapenem were all 100.0%; relatively high susceptibility rates were also observed for ceftazidime, ciprofloxacin, and cefepime, at 97.6, 95.2, and 95.0%, respectively. In addition, the susceptibility rates to ampicillin and ampicillin/sulbactam were 85.7 and 90.5%, respectively. In contrast, *Proteus mirabilis* still exhibited a certain degree of resistance to some agents, with the highest resistance rate observed for trimethoprim-sulfamethoxazole (34.2%), followed by ceftriaxone (14.6%) and ampicillin (14.3%). The resistance rates to cefepime, ciprofloxacin, and ceftazidime were 5.0, 4.8, and 2.4%, respectively. The intermediate rates for ampicillin/sulbactam and ceftriaxone were 9.5 and 2.5%, respectively.

**Table 7:** Antimicrobial susceptibility profiles of *Proteus mirabilis* to commonly used antimicrobial agents.

Antimicrobial agents	Resistance rate (%)	Susceptibility rate (%)	Intermediate rate (%)
Amikacin	0.0	100.0	0.0
Amoxicillin/clavulanic acid	0.0	100.0	0.0
Ampicillin	14.3	85.7	0.0
Ampicillin/sulbactam	0.0	90.5	9.5
Piperacillin/tazobactam	0.0	100.0	0.0
Ceftriaxone	14.6	82.9	2.5
Cefepime	5.0	95.0	0.0
Ertapenem	0.0	100	0.0
Ciprofloxacin	4.8	95.2	0.0
Trimethoprim-sulfamethoxazole	34.2	65.8	0.0
Ceftazidime	2.4	97.6	0.0

## 4 Discussion

This study systematically analyzed the composition of major bacterial isolates, their age and departmental distribution, and the antimicrobial susceptibility profiles of common bacteria based on vaginal discharge culture data collected from hospitalized patients at a tertiary maternal and child healthcare hospital in Shenzhen between 2021 and 2025. The results showed that vaginal isolates in this hospital were predominantly Gram-positive bacteria, among which *Streptococcus agalactiae* was overwhelmingly dominant, followed by *Escherichia coli* and *Klebsiella pneumoniae subsp. pneumoniae*. The isolates were mainly detected in specimens from the obstetrics department and from women aged 25–34 years. These findings indicate that *Streptococcus agalactiae* and common members of the Enterobacteriaceae family are bacterial species that warrant particular attention in vaginal specimens from hospitalized patients in this institution. At the same time, the marked differences in antimicrobial susceptibility profiles among bacterial species suggest that empirical antimicrobial therapy should be guided by the specific bacterial isolate identified and by local antimicrobial susceptibility surveillance data.

In the present study, *Streptococcus agalactiae* was the predominant isolate and was mainly detected in specimens from obstetric inpatients, which is consistent with the patient population typically served by maternal and child healthcare specialty hospitals. Although the study population was not categorized by pregnancy status, the predominance of obstetric specimens provides relevant perinatal clinical context rather than a pregnancy-status-specific subgroup analysis. *Streptococcus agalactiae* has long been recognized as an important colonizer during the perinatal period and as a major pathogen associated with early-onset neonatal infection (Arsenijevic et al., 2025; Manuel et al., 2025; Young et al., 2026). In recent years, increasing attention has been paid to the value of continuous surveillance of *Streptococcus agalactiae* colonization in the reproductive tract of pregnant women. As one of the major pathogens responsible for early-onset neonatal sepsis, the risk of maternal–infant vertical transmission of *Streptococcus agalactiae* remains a major focus of perinatal prevention and control (Delara et al., 2023; Micoli et al., 2026). Previous studies have shown that although intrapartum prophylactic strategies can substantially reduce the burden of early-onset *Streptococcus agalactiae* infection (Nusman et al., 2023), isolates of *Streptococcus agalactiae* from vaginal discharge specimens of pregnant women in China still exhibit high resistance rates to multiple non-β-lactam agents, particularly erythromycin, clindamycin, levofloxacin, and tetracycline. The findings of the present study are consistent with this overall trend, showing that *Streptococcus agalactiae* remained highly susceptible to penicillin, ampicillin, and vancomycin, whereas resistance to clindamycin and tetracycline was markedly elevated (Chen et al., 2024). These findings suggest that β-lactam agents should remain the first-line choice for perinatal prevention and treatment. For patients with penicillin allergy, clindamycin should not be used empirically without susceptibility evidence whenever possible, and individualized therapy based on antimicrobial susceptibility results is preferable.

Although *Klebsiella pneumoniae subsp. pneumoniae* and *Proteus mirabilis* accounted for a lower proportion of isolates than *Streptococcus agalactiae* and *Escherichia coli*, their resistance patterns also deserve attention. In the present study, the extended-spectrum β-lactamase positivity rate of *Klebsiella pneumoniae subsp. pneumoniae* was 31.8%, with relatively high resistance to ampicillin/sulbactam, ceftriaxone, and ciprofloxacin, whereas susceptibility to amikacin, carbapenems, and tigecycline remained high. *Proteus mirabilis*, by contrast, remained generally susceptible to most tested agents, although resistance to trimethoprim-sulfamethoxazole was relatively prominent. Compared with reports from the Eritrean National Health Laboratory and from women with abnormal vaginal discharge in Uganda, substantial differences were observed across regions in the predominant bacterial species and in specific resistance rates (Ahabwe et al., 2023; Hussein et al., 2024). However, one relatively consistent finding is that Gram-negative bacilli commonly show resistance to ampicillin, trimethoprim-sulfamethoxazole, tetracycline, and certain cephalosporins, while generally retaining good susceptibility to amikacin and some broad-spectrum reserve agents. These differences may reflect regional variation in antimicrobial use patterns and institutional background, and they also indicate that resistance data for vaginal discharge–related bacterial isolates cannot be directly extrapolated from other regions or from other types of healthcare institutions.

Among Gram-positive cocci, in addition to *Streptococcus agalactiae*, *Staphylococcus aureus* and *Enterococcus faecalis* were detected less frequently but still provided important clinical signals. In the present study, *Staphylococcus aureus* showed a high resistance rate to penicillin, and the cefoxitin screening positivity rate was 22.2%, suggesting the presence of a certain proportion of methicillin-resistant strains. However, susceptibility to vancomycin, linezolid, teicoplanin, and gentamicin remained high. Recent studies on lower genital tract colonization with *Staphylococcus aureus* in women have shown that methicillin-resistant *Staphylococcus aureus* colonization is not uncommon in some regions and may be associated with neonatal exposure risk (Ahabwe et al., 2023; Rallis et al., 2025). With regard to *Enterococcus faecalis*, recent studies on vaginal enterococci in pregnant women have shown substantial diversity in resistance profiles, particularly with respect to tetracyclines, macrolides, and even, in some cases, the carriage of oxazolidinone resistance genes (Xie Y. et al., 2025; Xie Z. et al., 2025). In our data, *Enterococcus faecalis* showed relatively high resistance to tetracycline and erythromycin, but remained susceptible to ampicillin, penicillin, vancomycin, and linezolid, with no evident glycopeptide or oxazolidinone resistance detected overall. To some extent, this suggests that the resistance burden of relevant strains in our institution remains manageable. Nevertheless, because the number of *Enterococcus faecalis* isolates in this study was limited, these findings still require validation in larger sample sets.

The present study also showed that isolates were mainly detected in women aged 25–34 years, and that the vast majority were from the obstetrics department. This distribution is closely related to the patient structure of maternal and child healthcare specialty hospitals and also indirectly indicates that perinatal women remain a key population for surveillance of vaginal isolates and for optimization of empirical therapy. Hormonal changes during pregnancy, reshaping of the vaginal micro-ecology, hospitalization, and perinatal procedures may all influence the isolate composition and resistance-related characteristics of vaginal bacterial isolates (Doroftei et al., 2023; Feduniw et al., 2025; Gerede et al., 2024; Styczynski et al., 2024). In addition, extensive perinatal exposure to antimicrobial agents may further drive local selection pressure for resistance (Winteler et al., 2025). In recent years, studies on perinatal antimicrobial stewardship and early-onset neonatal infection management have emphasized the need to balance risk stratification, microbiological evidence, and drug safety as much as possible, in order to avoid excessive empirical antimicrobial use. Taken together with the findings of the present study, the management of vaginal discharge–related bacterial infections in maternal and child healthcare specialty hospitals should not rely solely on empirical coverage, but should instead depend more heavily on stratified treatment strategies supported by local bacterial isolate profiles and resistance surveillance. Because pregnancy status and marital status were not included as subgroup variables, the present findings should be interpreted mainly according to patient age and clinical department.

Several limitations of this study should be acknowledged. This was a single-center retrospective analysis of hospitalized patients, and the findings may not be fully generalizable to outpatient populations or other regions. In addition, detailed clinical information, including symptoms and specific indications for vaginal swab collection, was not consistently available in the laboratory information system; therefore, we could not determine whether all specimens were collected for symptomatic evaluation or routine screening. Only bacteria recovered by culture were included, whereas other common pathogens related to abnormal vaginal discharge, such as anaerobes, mycoplasma, chlamydia, trichomonads, and fungi, were not assessed. Moreover, the number of isolates for some species, including *Enterococcus faecalis* and *Staphylococcus aureus*, was limited, and the corresponding resistance data should therefore be interpreted cautiously. Further studies integrating multicenter datasets, clinical outcome information, and molecular epidemiological approaches are warranted to better characterize the evolving distribution and resistance patterns of vaginal bacterial isolates in maternal and child healthcare settings.

## 5 Conclusion

Bacterial isolates from vaginal discharge specimens in hospitalized patients at this tertiary maternal and child healthcare hospital were dominated by *Streptococcus agalactiae* and common Enterobacteriaceae, with distinct antimicrobial susceptibility profiles across species. ESBL-positive *Escherichia coli* and *Klebsiella pneumoniae subsp. pneumoniae* isolates, together with cefoxitin screening-positive *Staphylococcus aureus* isolates, were identified, underscoring the need for continuous local surveillance of bacterial isolate distribution and antimicrobial susceptibility profiles in maternal and child healthcare settings. These findings may help refine empirical antimicrobial therapy and support antimicrobial stewardship, particularly among obstetric patients and women of reproductive age.

## Data Availability

The raw data supporting the conclusions of this article will be made available by the authors, without undue reservation.
